# Hypertensive Pressure Mechanosensing Alone Triggers Lipid Droplet Accumulation and Transdifferentiation of Vascular Smooth Muscle Cells to Foam Cells

**DOI:** 10.1002/advs.202308686

**Published:** 2023-12-25

**Authors:** Pamela Swiatlowska, William Tipping, Emilie Marhuenda, Paolo Severi, Vitalay Fomin, Zhisheng Yang, Qingzhong Xiao, Duncan Graham, Cathy Shanahan, Thomas Iskratsch

**Affiliations:** ^1^ School of Engineering and Materials Science Queen Mary University of London London E1 4NS UK; ^2^ Department of Pure and Applied Chemistry University of Strathclyde Glasgow G1 1QA UK; ^3^ Department of Translational Medicine Laboratory for Technologies of Advanced Therapies (LTTA) University of Ferrara Ferrara 44121 Italy; ^4^ Y.ai New York 10037 USA; ^5^ William Harvey Research Institute Queen Mary University of London London EC1M 6BQ UK; ^6^ School of Cardiovascular Medicine and Sciences King's College London London SE5 9NU UK

**Keywords:** atherosclerosis, foam cells, mechanosensing, pressure sensing, vascular smooth muscle cells

## Abstract

Arterial Vascular smooth muscle cells (VSMCs) play a central role in the onset and progression of atherosclerosis. Upon exposure to pathological stimuli, they can take on alternative phenotypes that, among others, have been described as macrophage like, or foam cells. VSMC foam cells make up >50% of all arterial foam cells and have been suggested to retain an even higher proportion of the cell stored lipid droplets, further leading to apoptosis, secondary necrosis, and an inflammatory response. However, the mechanism of VSMC foam cell formation is still unclear. Here, it is identified that mechanical stimulation through hypertensive pressure alone is sufficient for the phenotypic switch. Hyperspectral stimulated Raman scattering imaging demonstrates rapid lipid droplet formation and changes to lipid metabolism and changes are confirmed in ABCA1, KLF4, LDLR, and CD68 expression, cell proliferation, and migration. Further, a mechanosignaling route is identified involving Piezo1, phospholipid, and arachidonic acid signaling, as well as epigenetic regulation, whereby CUT&Tag epigenomic analysis confirms changes in the cells (lipid) metabolism and atherosclerotic pathways. Overall, the results show for the first time that VSMC foam cell formation can be triggered by mechanical stimulation alone, suggesting modulation of mechanosignaling can be harnessed as potential therapeutic strategy.

## Graphical abstract

Hypertensive pressure dependent vascular smooth muscle cells foam cell formation. Pressure leads to opening of Piezo1 channels at the outer, or inner nuclear membrane (ONM, INM). Calcium recruits cPLA2 to the INM, where it leads to cleavage of arachidonic acid (AA) and lipid droplet formation. Downstream of AA processing, reactive oxygen species (ROS) lead to epigenetic changes.

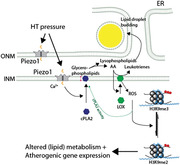

## 1 Introduction

Vascular smooth muscle cells (VSMCs) are the most abundant cells in the arterial wall. In a healthy artery they are contractile and found in the media layer. However, different stimuli cause a loss of the contractile phenotype, migration into the intima layer and further phenotypic switching.^[^ ^1^ ^]^ During the process, termed diffuse intimal thickening (DIT), the intimal VSMCs are proliferating, degrading the collagenous extracellular matrix (ECM) and secreting proteoglycans that bind to apolipoprotein B (apoB)‐containing lipoproteins in the subendothelial space. In the intima the lipid pools undergo oxidation and other modifications. This leads to the recruitment of circulating, or proliferation of tissue‐resident macrophages, an inflammatory response, and phagocytosis of lipids. This transition to so called foam cells is a critical part of the pathological intimal thickening (PIT) phase of early atherosclerosis. After undergoing apoptosis and secondary necrosis, foam cells release further inflammatory molecules, eventually leading to fibrous cap formation.

While foam cells are often simply regarded as lipid‐filled macrophages ,^[^ ^2^ ^]^ autopsy studies in the 1980s already suggested that VSMCs also start accumulating cholesterol inside of the cytoplasm in lipid droplets and are a major contributor to the cholesterol overloaded foam cell population.^[^ ^3^ ^]^ Because in contrast to humans, most common animal models for atherosclerosis lack a noteworthy accumulation of VSMCs in the intima layer, atherosclerosis research focused largely on macrophage‐derived foam cells and the contribution of VSMCs to foam cell populations remained unclear. Nevertheless, over the last decades studies demonstrated that VSMCs can express Class A scavenger receptor (SR‐A) after oxidative stress treatment and through atherogenic diet in rabbits in vivo, leading to lipid accumulation^[^ ^2^ ^]^; VSMC can accumulate lipids when cultured in adipocytic differentiation media through liver x receptor pathways^[^ ^4^ ^]^; bone marrow stem cells derived VSMCs, when treated with oxidized low‐density lipoprotein (oxidized LDL), increased SR‐A and reduced ATP‐binding cassette transporter A1 (ABCA1) and caveolin‐1 protein expression, all hallmarks of foam cell formation.^[^ ^5^ ^]^ Especially the reduction in ABCA1 suggested reduced cholesterol efflux to be in part responsible for the lipid accumulation. However, in contrast to the stem cell derived VSMCs, in rabbit only native and not modified LDL was able to stimulate foam cell formation.^[^ ^6^, ^7^, ^8^ ^]^ Co‐expression analysis of smooth muscle α‐actin and the macrophage marker CD68 further indicated that a larger proportion of foam cells (∼50%) was indeed derived from VSMCs, with an disproportionate contribution to disease progression through progressively reduced ABCA1 levels, suggesting a reduced lipid flux compared to macrophage foam cells.^[^ ^9^ ^]^ More recent lineage tracing studies could also recapitulate this in apoE‐deficient mice, where VSMCs with reduced ABCA1 expression were found to made up the majority of foam cells,^[^ ^10^ ^]^ suggesting that ABCA1 might be a key regulator of lipid accumulation and phenotypic switching. In addition, knock out of Krüppel‐like factor 4 (KLF4) in VSMCs reduced the number of macrophage‐like cells and increased plaque stability, suggesting involvement in foam cell formation. KLF4 overexpression inhibits expression of contractile markers and levels of KLF4 are elevated in response to platelet‐derived growth factor signaling and counteracted by transforming growth factor β1 signaling or retinoic acid. KLF4 is further regulated through miRNAs in both directions (increased contractility with miR‐1, increased phenotypic switching with miR‐143/145, overall placing KLF4 as a master‐regulator of macrophage/foam cell transition.^[^ ^11^ ^]^ However, KLF4 does not regulate ABCA1 expression,^[^ ^12^ ^]^ suggesting several distinct mechanisms might be regulating foam cell formation and details of VSMC foam cell formation making this area an active topic for research.

Mechanical sensing and signalling play a key role in shaping many pathophysiological processes, including in the cardiovascular system.^[^ ^13^, ^14^ ^]^ In addition to chemical signals (e.g., from the endothelial cells, or through lipid interactions), we recently demonstrated that VSMCs respond to mechanical stimuli as well.^[^ ^15^ ^]^ Especially, hypertensive pressure and extracellular matrix compliance, mimicking the diseased intima can stimulate a change in the cytoskeleton and cell morphology, remodelling of the extracellular matrix, and changes in the protein content with differential regulation of proteins involved in atherosclerosis, cytoskeletal regulation and podosome formation.^[^ ^15^ ^]^ Here, we find in bioinformatic analysis a likely regulation through adipogenic pathways. Indeed, we observe that hypertensive pressure stimulated VSMC (A7r5 and primary human and rat) display major hallmarks of foam cell transition, including lipid droplet accumulation, increased levels of CD68 and reduced levels of ABCA1. We further find this is dependent on calcium transients downstream of Piezo1 and its activation can induce lipid droplet accumulation in cultured VSMCs. Mechanistically, we find that Piezo1 activation leads to changes in nuclear calcium uptake, reactive oxygen signalling, and downstream Histone 3 lysine 9 trimethylation (H3K9me3). Immunohistochemistry, biochemistry, electron microscopy and CUT&Tag experiments confirm reduced H3K9me3 and reduced repression of genses related to cell cycle regulation, metabolism and especially lipid metabolism, as well as atherosclerotic regulators. Together our data demonstrates for the first time that mechanical stimuli alone are sufficient to induce a foam cell phenotype and outline the molecular pathway of the phenotypic switching.

## 2 Results

### 2.1 Piezo1 Localization in Vascular Smooth Muscle Cells

**Figure 1:**
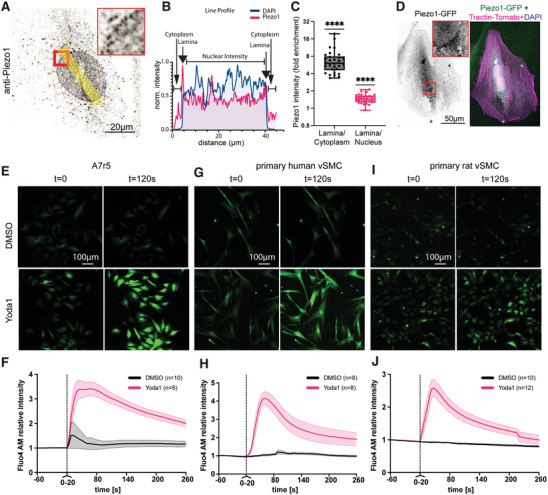
Piezo1 localises to the VSMC nuclear lamina and controls calcium levels. Immunofluorescent micrographs shows enrichment of Piezo1 at the nuclear membrane of A7r5 VSMCs (A–C). A) Anti‐piezo1 staining shows a dotted appearance around the nuclear membrane (zoom in inset). B) Example line profile (line indicated in A) shows enrichment at the edges. C) Quantification of enrichment against cytoplasm and nuclear intensity. **** *P*<0.0001 from one‐sample t‐test, compared to a value of 1, i.e., no enrichment. D) The nuclear localisation is further confirmed using a Piezo1‐GFP transfection (zoom in inset); here co‐transfected with Tractin‐Tomato. E–J) Yoda1 treatment leads to rapid Ca^2+^ transients in A7r5 (E,F), primary human (G,H) and primary rat (I,J) VSMCs. Data from three independent repeats with *n* = 30–45 cells.

We previously studied vascular mechanosensing in response to pathophysiological arterial stiffness and hypertensive pressure and found that distinct mechanosensing pathways downstream of stiffness and pressure sensing converged to define the mechanoresponse.^[^ ^15^ ^]^ Especially, we found elevated Ca^2+^ levels in VSMCs following hypertensive pressure application (200/120 mmHg), with especially dramatic calcium increases in the nucleus, but the source of the calcium remained unclear. Piezo1 is a known Ca^2+^‐permeable channel activated by mechanical stimuli that has been described in VSMCs previously, thus we hypothesized that Piezo1 was mediating the detected pressure response. Indeed, super‐resolution spinning disc confocal microscopy indicated the presence of Piezo1 in punctate localisation along the cell membrane, inside the cytoplasm and particularly pronounced in the nucleus and the nuclear membrane (**Figure** **1**A–C). Quantification of the staining pattern indicated a ≈sevenfold enrichment in the nuclear lamina compared to the cytoplasm and a ≈50% enrichment in the lamina compared to the nuclear staining.

While nuclear localization of Piezo1 was described before,^[^ ^16^, ^17^ ^]^ we verified the nuclear localization and antibody specificity using transfection with a human Piezo1‐GFP plasmid (Figure 1D).

To assess the physiological significance of Piezo1 activation, we used the Piezo1 agonist, Yoda1 on A7r5, primary human and primary bovine VSMC that were pre‐incubated with Fluo‐4 AM as Ca^2+^ indicator. After treatment, an immediate response was observed with consistent Ca^2+^ increase in A7r5, primary human, and bovine cells (Figure 1E‐J). We again found a particularly strong Ca^2+^ increase in the nucleus that was absent in the DMSO treated control groups. The response was further suppressed by Dooku1, the antagonist of Yoda1‐induced Piezo1 channels (Figure S1, Supporting Information),^[^ ^18^ ^]^ indicating that Piezo1 can mediate the mechanosensitive Ca^2+^ response in VSMCs.

### 2.2 Piezo1 Stimulation Supports a Foam Cell‐Like Phenotype

**Figure 2:**
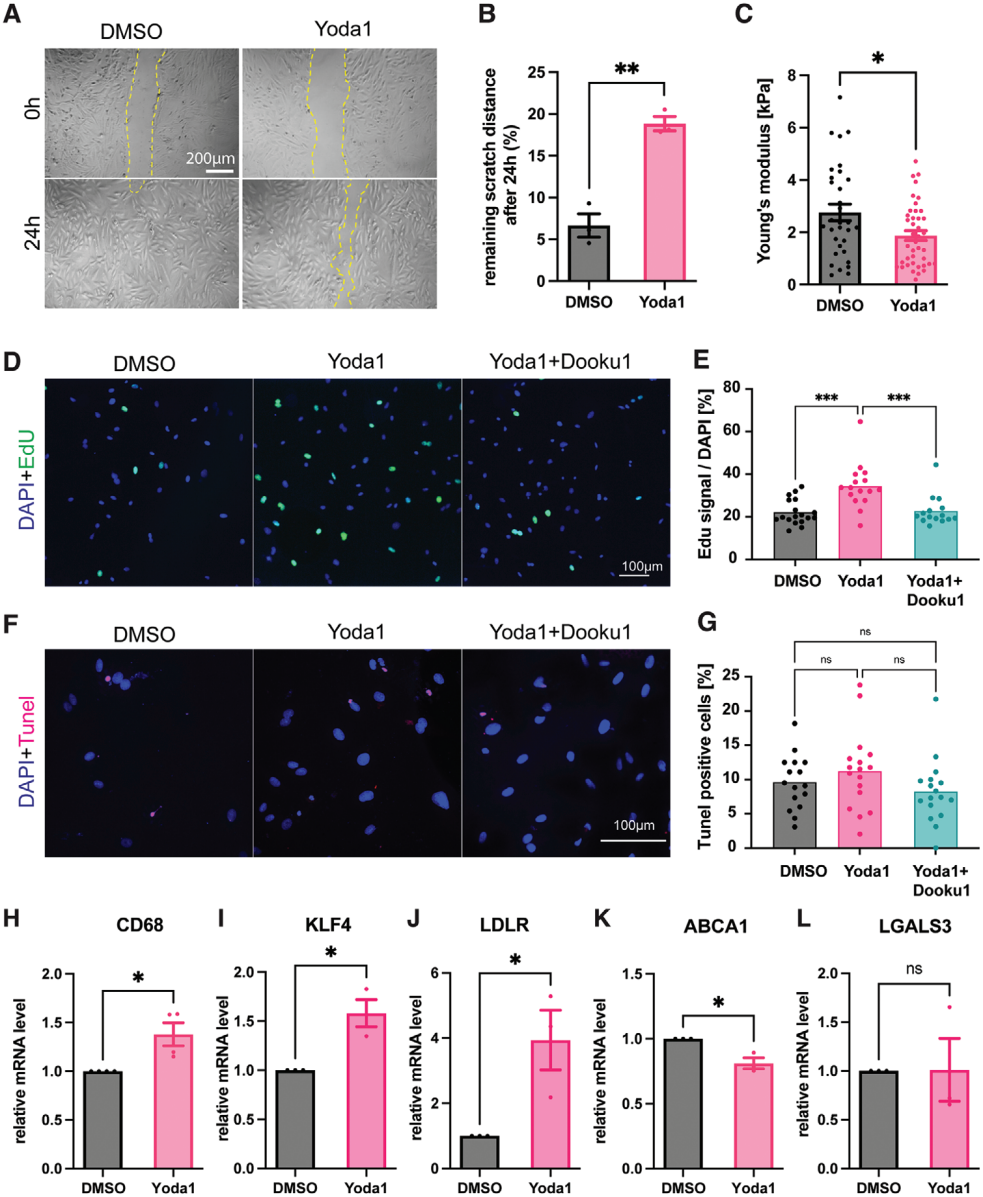
Chronic Yoda1 treatment supports transition to a foam cell phenotype. A‐B) Wound scratch assay shows reduced migration of Yoda1 treated A7r5 cells. C) Nanoindentation indicates a lower Young's Modulus after Yoda1 treatment. D,E) Click‐EdU assay indicates higher cell proliferation following Yoda1 incubation, which is reversed after simultaneous Dooku1 treatment. F,G) TUNEL staining indicates no significant changes to apoptosis after 8 h Yoda1 treatment. H–L). qPCR testing indicate increased CD68 (H), KLF4 (I), LDLR (J) and lower ABCA1 (K) transcription after 8‐hour Yoda1 treatment, while LGALS3 (L) showed no change at this time point. p‐values from unpaired two‐tailed t‐tests (B,C, H–L), or one‐way ANOVA with Tukey correction for multiple comparisons (E,G): * *p*<0.0332, ** *p*<0.0021, *** *p*<0.0002.

We next wanted to test the functional implications of Piezo1 stimulation. First, we investigated the effect of Piezo1 activation on VSMC migration and proliferation, as hallmarks of phenotypic switching. Using an i*n vitro* wound scratch assay, we observed that A7r5 cell motility was significantly decreased at 24 h after Yoda1 treatment (**Figure** **2**A,B). Additionally, we found a reduced cellular Young's modulus after Yoda1 treatment, consistent with the phenotypic transformation and previous observations after hypertensive pressure stimulation (Figure 2C).^[^ ^15^ ^]^ Using an EdU‐Click Assay we further found significantly increased proliferation after Yoda1 treatment, when compared to the DMSO control, which was reversed when simultaneously treated with Dooku1 (Figure 2D,E). In contrast, a terminal deoxynucleotidyl transferase dUTP nick end labeling (TUNEL) assay indicated no significant changes to apoptosis after Yoda1 treatment (Figure 2F,G) or hypertensive pressure stimulation (Figure S2, Supporting Information). qPCR testing after 8 h Yoda1 treatment indicated increased CD68, KLF4, low density lipoprotein receptor (LDLR), and decreased ABCA1 levels, consistent with a macrophage‐like or foam‐cell phenotype (Figure 2H–K). In contrast, Galectin‐3 (LGALS3), linked to an osteogenic state^[^ ^19^ ^]^ was not significantly altered at this point (Figure 2L). Intriguingly, bioinformatic analysis of our previous proteomics data, using the causal reasoning algorithm, identified several molecules related to foam cell formation and lipid metabolism as potential upstream regulators of the changes in the proteome between mechanically stimulated and control cells (Figure S3, Supporting Information) ,^[^ ^15^, ^20^ ^]^ further supporting the notion that mechanical stimulation would be sufficient to induce VSMC foam cell formation.

### 2.3 Acute Piezo1 Activation Leads to Rapid Lipid Droplet Formation

**Figure 3:**
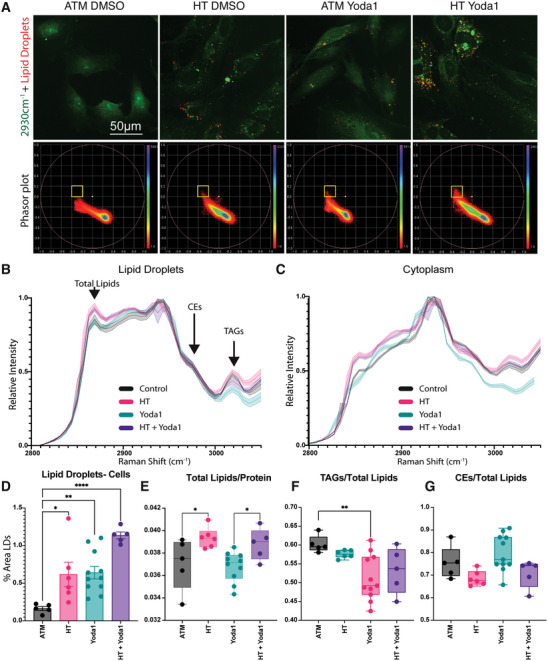
Hyperspectral stimulated Raman Scattering (hsSRS) indicates lipid droplet accumulation and lipid metabolic changes after pressure and Yoda stimulation. A,B) Acute Piezo1 activation and HT pressure stimulation leads to lipid accumulation in A7r5 cells. A) Lipid droplet segmentation from phasor blots (bottom row) overlaid in red over 2930cm‐1 image for overall cell outlines (green); B,C) hsSRS shows increase in total lipids after HT pressure and reduction in TAGs after Yoda1 treatment in lipid droplets (B) and cytoplasm (C). D) Quantification of area ratio of lipid droplets; E) Quantification of ratios of Total Lipids compared to Proteins, F) TAGs compared to total Lipids, and G) cholesterol esters (CEs) compared to total lipids. p‐values from one‐way ANOVA with Dunnet correction for multiple comparisons (against ATM): * *p*<0.0332, ** *p*<0.0021, **** *p*<0.0001. Only significant comparisons are shown.

Since we found expression of macrophage markers, we next wanted to test for changes in the lipid content and lipid droplet formation. Stimulated Raman scattering (SRS) offers label‐free, non‐destructive testing of cellular biomolecules. More recently, the development of hyperspectral SRS (hsSRS) enabled the chemical mapping of molecules with similar Raman spectra, such as cholesterol esters (CEs) or Triacylclycerides (TAGs), while the implementation of spectral phasor analysis of the hyperspectral datasets allows the differentiation of discrete cellular compartments, such as nuclei, membrane, cytoplasm or lipid droplets.^[^ ^21^, ^22^ ^]^ We therefore employed hsSRS for the analysis of lipid droplet content and chemical signatures after pressure treatment and/or Piezo1 activation. Indeed, consistent with a foam‐cell phenotype, we found clear and rapid increases in lipid droplet formation after short‐term hypertensive pressure stimulation (2 h) or Piezo1 activation (starting from 30 min, **Figure** **3**A–D). The lipid droplet formation was further enhanced through combined pressure stimulation and Yoda1 treatment (2 h). Yoda1 dependent lipid droplet formation and its reversal through simultaneous treatment with Dooku1 was additionally confirmed using a lipid dye (Lipidspot, Figure S4, Supporting Information). Closer inspection of the lipid composition suggested important differences between pressure and Yoda1 treated samples, namely an overall increase in lipids^[^ ^22^ ^]^ in lipid droplets and cytoplasm of pressure treated cells, which was absent in Yoda1 treated cells (Figure 3B,C). This result indicated that Piezo1 independent mechanisms were leading to additional lipid uptake in pressure stimulated cells. Moreover, we detected a reduction of TAGs (peak at 3018cm^−1^) in Yoda1 treated cells. Because TAG synthesis is the preceding step for lipid droplet formation,^[^ ^23^ ^]^ this surprisingly suggested that the rapid lipid droplet formation, without simultaneous lipid uptake, was leading to a depletion in the bioavailable lipids. In agreement with this, we found a dramatic increase in lipid droplet formation and overall increase in lipids (in cytoplasm and lipid droplets) after combined pressure and Yoda1 treatment and no significant reduction in TAGs (Figure 3B,C). No changes were found for cholesterol esters (peak/shoulder at 2962 cm^−1^). Consistent with the hsSRS data, we found no change to levels of class B scavenger receptor CD36 and even a reduction in levels of class A macrophage scavenger receptor 1 (MSR1), both linked to modified lipid and especially oxidized LDL uptake (Figure S5, Supporting Information).^[^ ^24^ ^]^

Similar changes were found in a mouse model for intima hyperplasia after carotid artery ligation, where hsSRS could identify the extracellular matrix, cytoplasm, and lipid droplet signatures (Figure S6, Supporting Information), demonstrating the outstanding capabilities of this method and potential applications in diagnostics. Lipid droplets were dramatically increased in intimal hyperplasia and especially in the phenotypically switched cells toward the luminal side.^[^ ^15^ ^]^ Similar to Yoda1 treated cells and consistent with a lack of hypertensive pressure in this model, we found here reduced levels of TAGs, but no change in overall lipids. In contrast to our cell model we found however increased levels of cholesterol esters, related to the lipid retention in this model.^[^ ^25^ ^]^

Overall, these results demonstrate that Piezo1 activation alone can lead to a rapid stimulation of lipid droplet formation, while pressure stimulation additionally leads to an uptake of extracellular lipids.

### 2.4 Yoda1 Stimulation Promotes Foam Cell‐Like Phenotype

**Figure 4:**
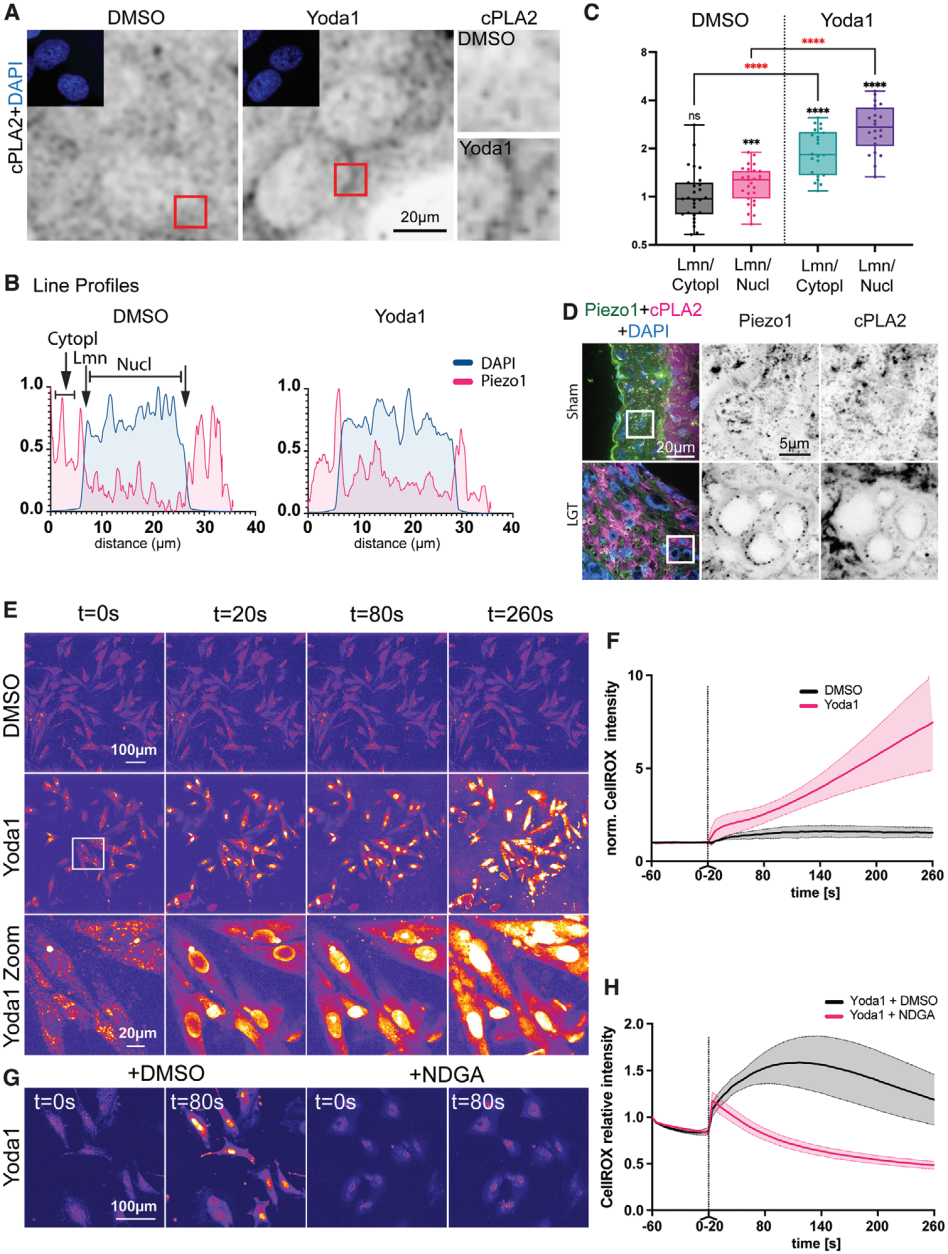
cPLA2 accumulation and ROS upregulation after Piezo1 activation. A) Immunofluorescent micrographs demonstrating cPLA2 accumulation following Yoda1 treatment. B) example line profiles over nucleus. C) Quantification of enrichment against cytoplasm and nuclear intensity. Pooled data from three independent repeats. *** *p*<0.0002, **** *p*<0.0001; p‐values from one‐sample t‐test compared to 1 (black, i.e., 1 = no enrichment) or unpaired two‐tailed t‐test (red). D) Immunofluorescent staining of mouse tissues after artery ligation or sham control. E–H) A7R5 cells incubated with CellROX dye. Acute Piezo1 activation leads to increased ROS production (E, F), which is blocked by simultaneous LOX inhibition through NDGA treatment (G, H). Data from three independent repeats with *n* = 25–40 cells.

Lipid droplet accumulation is a key feature of foam cells with cytosolic phospholipase A_2_ (cPLA2) being involved in the formation of the lipid‐based organelles.^[^ ^23^, ^26^ ^]^ The action of cPLA2 leads to the formation of free fatty acids especially arachidonic acid (AA) and lysophospholipids from phospholipids, whereby lysophospholipids are critical for budding of lipid droplets from the endoplasmic reticulum (ER) membrane.^[^ ^23^, ^26^ ^]^ Ca^2+^ ions are the main activators of cPLA2, relocating active cPLA2 to the nuclear membrane.^[^ ^27^ ^]^ Indeed, immunofluorescent staining for cPLA2 indicated nuclear membrane accumulation after Piezo1 treatment (**Figure** **4**A–C). A similar nuclear re‐localization was also found in arterial tissues with intimal hyperplasia, but not in arteries from sham operated animals (Figure 4D). cPLA2 cleaves glycerophospholipids to form free AA, which is further metabolized by lipoxygenases (LOX) to form bioactive leukotrienes, which are mediators for intimal hyperplasia and atherosclerotic plaque formation.^[^ ^28^, ^29^ ^]^ The enzymatic processing of AA by LOX lead to reactive oxygen species (ROS) formation as a by‐product, which in turn increase the activity of cPLA2 in a feed‐forward loop.^[^ ^30^ ^]^ Moreover, high ROS levels stimulates foam cell formation.^[^ ^31^ ^]^ To test the possibility of Piezo1 and cPLA2‐dependent ROS accumulation in VSMCs, we incubated the cells with the CellROX, a live cell oxidative stress detector, and stimulated cells with Yoda1. A gradual increase in ROS was observed immediately after stimulation (Figure 4E,F). ROS was initially formed at the nuclear envelope and expanded further over the nucleus and subsequently the cytoplasm, consistent with an enzymatic reaction at the inner nuclear envelope. This was dependent on LOX, since it was inhibited by simultaneous incubation with the pan‐LOX inhibitor NDGA (Figure 4G,H).

Together this outlines a pathway of Piezo1 dependent mechanosensing leading to nuclear calcium increases, cPLA2 recruitment and LOX dependent formation of AA and leukotrienes, as well as ROS as a by‐product, which again amplifies the cPLA2 activity.

### 2.5 Piezo1‐Dependent Histone Epigenetic Changes

Foam‐cells exhibit a M2 macrophage‐like phenotype.^[^ ^32^, ^33^ ^]^ The very dynamic nature of the atherosclerotic lesion environment requires timely regulation of gene expression in all the cells. M2 macrophage demonstrate a specific epigenetic landscape. One of the characteristics is described by downregulation of histone 3 lysine 9 trimethylation (H3K9me3), a repressive histone mark.^[^ ^34^, ^35^, ^36^ ^]^

**Figure 5:**
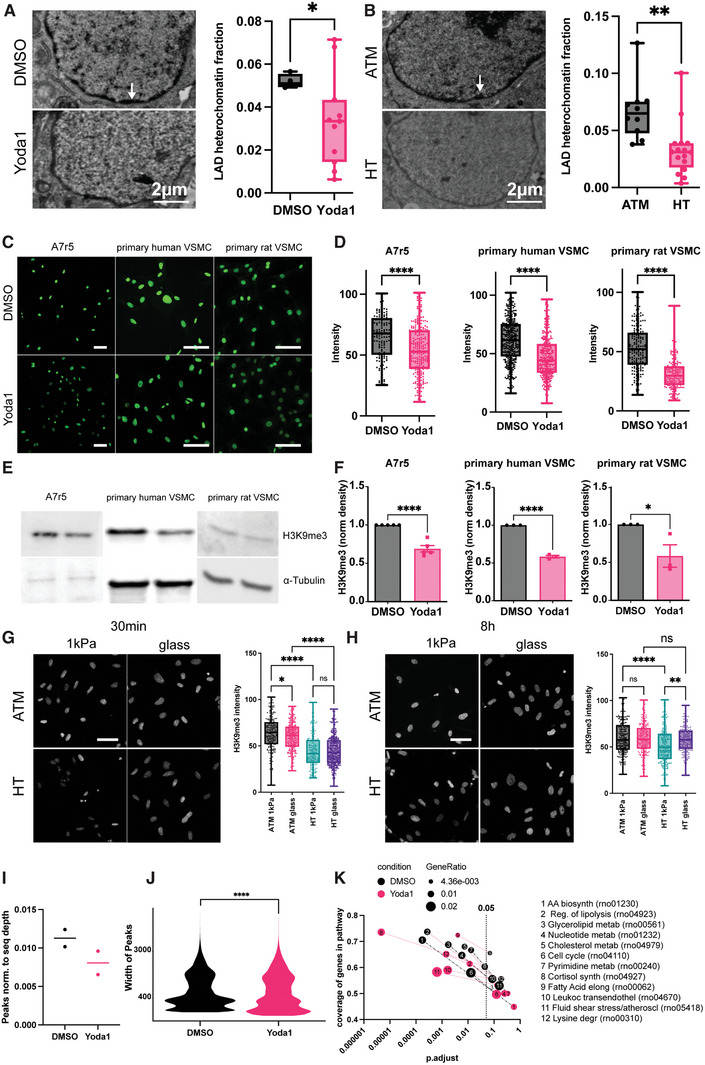
Hypertensive pressure and Piezo1 control epigenetic changes through H3K9me3. A,B) Piezo1 activation (A) and hypertensive pressure (B) decreases lamin‐associated domain heterochromatin in A7r5 cells, as assessed from transmission electron micrograph analysis. C,D) Decreased H3K9me3 histone modification level in A7r5, primary human and primary rat VSMC are detected on immunofluorescent staining and western blots (E,F, three independent repeats. N = 40‐90 cells). G) Acute hypertensive pressure reduces H3K9me3 level on 1 kPa and glass substrates, but fails to change the level on glass substrate at the chronic timepoint (H). I–K) CUT&Tag confirms a reduction in H3K9me3 peaks (I) and peak width (J). KEGG pathway analysis indicates changes to metabolism and cell cycle downstream of epigenetic regulation. * *p*<0.0332, ** *p*<0.0021, *** *p*<0.0002, **** *p*<0.0001; p‐values from unpaired two‐tailed t‐test (A,B,D,F, J) or one‐way ANOVA with Tukey correction for multiple comparisons. (G,H). Scale bars (C,G,H): 100 µm.

To test for potential changes to transcriptional repression, we first quantified the lamin‐associated domains (LAD) of heterochromatin. Based on transmission electron micrographs we identified less LAD‐heterochromatin following Yoda1 incubation (**Figure** **5**A) and a similar reduction was observed in cells subjected to hypertensive pressure (Figure 5B). To gain further insights into the histone methylation status, A7r5 cells, primary human and primary rat VSMC were subjected to a 30‐minute Yoda1 treatment. Confocal microscopy indeed revealed decreased H3K9me3 compared to control samples (Figure 5C,D), which was further confirmed by Western blot analysis (Figure 5E,F). In contrast, no changes were found for other histone marks, including histone 3 lysine 27 trimethylation (H3K27me3), histone 3 lysine 9 acetylation (H3K9ac), or histone 3 lysine 27 acetylation (H3K27ac) (Figure S7, Supporting Information).

Similar to Yoda1 treatment, acute, 30‐minute hypertensive pressure application decreased H3K9me3 on 1 kPa substrates compared to atmospheric pressure and identical result were observed on glass substrates (Figure 5G). Extended pressure application (8 h), again led to decreased H3K9me3 on 1 kPa substrates, but not on the stiff glass coverslips (Figure 5H), suggesting longer term adaptation due to stiffness sensing.

CUT&Tag epigenetic analysis of DMSO and Yoda1 treated A7r5 cells, performed with a H3K9me3 antibody consistently revealed a reduction in the peak numbers normalised to read depth (Figure 5I; Figure S8A, Supporting Information) and peak width (Figure 5J). To combine the replicates and limit the downstream analysis to the highly reproducible peaks we applied the Irreproducibility Discovery Rate (IDR) framework,^[^ ^37^ ^]^ resulting in 75557 and 62694 reproducible peaks for DMSO and Yoda1 respectively. Peak analysis suggested no noticeable difference in the localization of the peaks in respect to genic and intragenic regions (Figure S8B, Supporting Information). KEGG Pathway analysis of the identified peaks indicated overlapping pathways unrelated to VSMC function (Figure S8C,D and Table S1, Supporting Information;, i.e., neuronal pathways, bacterial and viral infection, cardiomyopathy and cancer pathways), consistent with a repressive histone mark in VSMCs. In addition to these, enrichment (i.e., repression) of several pathways was lost after Yoda1 treatment (Figure 5K). These included the pathways “cell cycle” (rno04110), “biosynthesis of amino acids” (rno01230), “nucleotide metabolism” (rno01232), “glycerolipid metabolism” (rno00561), “regulation of lipolysis in adipocytes” (rno04923), “pyrimidine metabolism” (rno00240), or “cholesterol metabolism” (rno04979), suggesting both metabolic changes related to increased cell growth/proliferation, as well as increased lipid metabolism, consistent with the foam cell phenotype (Tables S1 and S2, Supporting Information). Pathways that were enriched only after Yoda1 treatment included “fluid shear stress and atherosclerosis” (rno05418), “leukocyte transendothelial migration” (rno04670), “lysine degradation” (rno00310) and “fatty acid elongation” (rno00062) (Tables S2–S, 14, Supporting Information). Because an enrichment of this pathway in Yoda1 treated cells was potentially inconsistent with the phenotypic switching we looked at the differently regulated genes in the “fluid shear stress and atherosclerosis” pathway in further detail (Table S13, Supporting Information). This indicated several entries related to glutathione S‐transferase, whereby there seemed to be an isoform switch (isoforms omega1, omega 2, mu5 repressed in DMSO treated cells and isoforms alpha1, mu2, mu6‐like and microsomal glutathione S‐transferase 1 repressed in Yoda1 treated cells), potentially related to an adaptation to oxidative stress.^[^ ^38^ ^]^ Additionally we found peaks in Yoda1 treated cells for *arhgef2* (also known as GEF‐H1, an inhibitor of podosome formation^[^ ^39^, ^40^ ^]^), *calm1* (calmodulin, downregulation consistent with ABCA1 loss/foam cell formation),^[^ ^41^ ^]^ *acvr1* (activin A receptor, consistent with a loss of contractile phenotype),^[^ ^42^ ^]^ *dusp1* (also known as MKP1; expressed at atheroprotected regions^[^ ^43^ ^]^ and reduced levels associated with increased VSMC proliferation^[^ ^39^ ^]^). On the other hand, key pro‐atherosclerotic genes including *klf2* (Kruppel‐like factor 2), *sele* (E‐selectin), or *fos* (which is critical for foam cell formation and atherosclerosis^[^ ^44^ ^]^) all had identifiable H3K9me3 peaks and thus were repressed in DMSO treated cells only. Overall, the CUT&Tag experiment confirms an epigenetic regulation of VSMC phenotypic switching through regulation of metabolic pathways and atherosclerotic as well as foam cell gene regulation.

**Figure 6:**
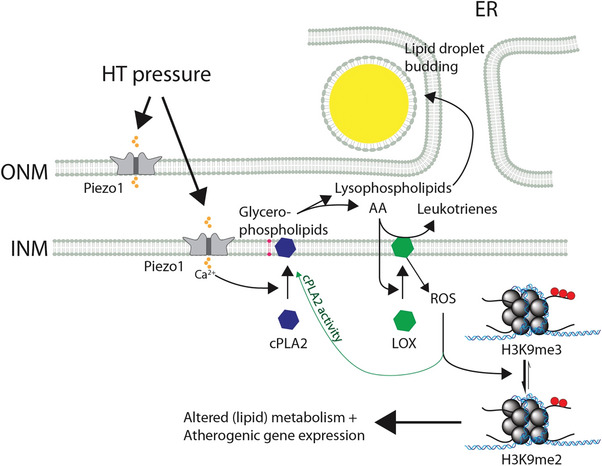
Model of hypertensive pressure dependent VSMC foam cell formation. HT pressure leads to opening of Piezo1 channels at the outer (ONM) and/or inner nuclear membrane (INM) and calcium influx into the nucleoplasm. Calcium leads to recruitment of cPLA2 to the INM, where it cleaves glycerophospholipids into lysophospholipids and free arachidonic acid (AA). Lysophospholipids stimulate lipid droplet budding from ER while AA stimulates translocation of lipoxygenases (LOX) from the nucleoplasm to the INM. LOX converts AA to leukotrienes and at the same time produces reactive oxygen species (ROS) as a by‐product. ROS increases cPLA2 activity and induces the demethylation of Histone 3 lysine 9, leading to transcription of genes involved in metabolism, lipid metabolism and atherosclerosis.

Together these results demonstrate that Piezo1 controls VSMC foam cell transition through short‐term effects (lipid droplet accumulation) as well as long‐term metabolic and epigenetic changes (see **Figure** **6** for model).

## 3 Discussion

Previous research has increasingly demonstrated the critical influence of VSMC derived foam cells in atherosclerotic disease progression. In addition to their relative proportion of the total foam cell pool, their reduced cholesterol efflux results in an overproportioned accumulation of lipids and resistance to atherosclerosis regression treatments.^[^ ^9^, ^45^ ^]^ However, the regulation of the foam cell transition remained elusive. Our previous research demonstrated the influence of hypertensive pressure and extracellular matrix compliance alone and especially in combination on VSMC morphology, cytoskeletal organization and function, including extra cellular matrix degradation. Proteomic analysis demonstrated a range of differentially regulated proteins with or without pressure stimulation. Moreover, we found pressure dependent Ca^2+^ transients that were leading to slingshot phosphatase activation, cofilin dephosphorylation and podosome formation.^[^ ^15^ ^]^ Here we find these Ca^2+^ currents are regulated through the Piezo1 channel and Piezo1 is indeed instrumental in regulating the foam cell transformation, as suggested by bioinformatics analysis, qPCR analysis for key foam cell markers, cell proliferation and migration analysis, cell mechanical analysis and hyperspectral Raman spectroscopy analysis.

The mechanosensitive Piezo channels have been previously described in VSMCs and associated with structural remodeling of small arteries in hypertensive conditions, through regulating transglutaminase activity.^[^ ^46^ ^]^ VSMC Piezo1 was further linked to  increased contraction and proliferation ^[^ ^17^, ^47^ ^]^ and apoptosis.^[^ ^48^ ^]^ Additionally, Piezo1 was upregulated in abdominal aortic aneurysms downstream of netrin‐1.^[^ ^49^ ^]^

Although the Piezo channels are generally targeted to the plasma membrane,^[^ ^50^, ^51^ ^]^ where they are localized in a curvature dependent way,^[^ ^52^ ^]^ localization to diverse intracellular membranous organelles, including sarcoplasmic and endoplasmic reticulum, mitochondria, Golgi has been reported before.^[^ ^16^, ^17^, ^53^ ^]^ In mitochondria, it regulates oxidative phosphorylation through cyclic AMP^[^ ^53^ ^]^ and can lead to mitochondrial‐dependent apoptosis after Ca^2+^ overload and ROS accumulation.^[^ ^48^ ^]^ The Piezo1 dependent release of Ca^2+^ from the ER/SR regulates pulmonary smooth muscle cell contraction and vasoconstriction^[^ ^17^ ^]^ or activation of calpain, integrins and epithelial adhesion.^[^ ^54^ ^]^ The localization to the Golgi is likely related to its posttranslational processing and especially glycosylation.^[^ ^55^ ^]^ While localization of Piezo1 to the nuclear envelope has been reported before, its functional implication remained unexplored.^[^ ^16^, ^17^ ^]^

Here we find that Piezo1 at the nuclear envelope senses hypertensive pressure, leading to nuclear Ca^2+^ spikes that indeed are further regulating nuclear cPLA2 accumulation and downstream activation of the AA pathway, including ROS increases at the nuclear membrane that only afterwards spread over the nucleoplasm and cytoplasm. The hsSRS data further indicated rapid lipid droplet formation after pressure stimulation and Piezo1 activation, suggesting a direct mechanism rather than a mechanism through gene regulation that is consistent with previous research linking cPLA2 and AA with lipid droplet formation.^[^ ^23^, ^26^, ^30^ ^]^ Lipid droplet (LD) formation is a step‐wise process, whereby TAGs first accumulate in the ER lumen, leading to a lipid lens formation after a critical threshold is reached. LDs grow in part through re‐localization of the TG synthesis machinery to the LD surface, followed by budding into the cytoplasm. LDs then can expand further through ripening or fusion .^[^ ^56^ ^]^ Our results point to a regulation of the budding and/or overall TAG homeostasis resulting in the increased LD formation. First, our data from hsSRS and lipid staining show localization of the LDs throughout the cytoplasm, and not restricted to the ER, suggesting that most LDs have indeed already budded. Second, we see a reduction in TAGs in both cytoplasm and LDs after Piezo1 stimulation suggests that rapid LD formation might lead to a depletion of the TAG pool, ruling out a regulation through increased TAG synthesis. Intriguingly however, there is no TAG depletion after pressure treatment, but conversely an overall increase in total lipids, suggesting a stimulation of lipid formation or uptake from the media in a pressure dependent but Piezo1 independent way. Thirdly, our data show increased cPLA2 localization to the nuclear envelope after Piezo1 activation. cPLA2 cleaves glycerophospholipids to generate free AA and lysophospholipids, whereby lysophospholipids induce budding of lipid droplets.^[^ ^23^, ^26^ ^]^

AA on the other hand acts on lipoxygenases (LOX) to induce their transition to the inner nuclear membrane where it co‐localizes with cPLA2 and catalyses the further processing of AA to leukotrienes, which again are critically involved in intimal hyperplasia and further atherogenic processes.^[^ ^28^, ^29^, ^57^, ^58^, ^59^ ^]^

The enzymes metabolising AA, especially LOX generate reactive oxygen species (ROS) as a by‐product, which we could indeed confirm, by observing an initial rapid increase of ROS at the inner nuclear membrane, which was blocked after treatment with a pan‐LOX inhibitor.^[^ ^30^, ^60^ ^]^ Interestingly, ROS further leads to an activation of cPLA2, suggesting a feed‐back mechanism driving the LD budding^[^ ^30^, ^61^ ^]^ (see also model in Figure 6).

At the same time ROS has been further linked to epigenetic changes and especially changes to histone methylation, which we find here as well.^[^ ^62^ ^]^ Histone methyl transferases require S‐adenosylmethionine as substrate, which again has been reported to be sensitive to ROS. Our attempts to measure SAM levels via ELISA assay suggested no significant difference (data not shown). Nevertheless, the experiment might have suffered from insufficient sensitivity of this test, as we found high variability in the outcomes and two out of three experimental repeats indeed suggested a ≈40–50% reduction in SAM levels. On the other hand, expression levels of a range of histone methyltransferases and demethylases are regulated through ROS,^[^ ^62^ ^]^ although the rapid change in H3K9me3 levels (30 min) might not be fully consistent with the timescale of transcriptional changes. Lastly, KDM lysine histone demethylases have been shown to be reactive to nitric oxide in vitro, albeit this led to a deactivation and thus is inconsistent with reduced H3K9me3 levels in our hands.^[^ ^63^, ^64^ ^]^

Along with the Piezo1/pressure‐dependent downregulation of H3K9me3 that effectively results in decreased levels of heterochromatin, we also observed reduced Young's modulus, measured just above the nucleus after Yoda1 treatment (reported here) or pressure stimulation (reported previously).^[^ ^15^ ^]^ These results are in line with the dogma that chromatin compactness is one of the components contributing to the overall nuclear stiffness.^[^ ^65^ ^]^

The atherosclerotic environment is characterized by altered mechanical properties. Foam cells and macrophages respond to changes to local stiffness by a more efficient, oxidized lipid uptake on soft substrates.^[^ ^66^ ^]^ To verify whether M2 characteristic low H3K9me3 level is also observed in the soft milieu, we employed 1 kPa PDMS substrate, that corresponds to the regional plaque stiffness, along with pressure stimulation.^[^ ^15^, ^34^, ^35^ ^]^ Similarly, short term pressure application results in H3K9me3 change on both, 1 kPa and glass substrates, but not on glass followed by an 8‐hour stimulation. Previously observed, VSMC obtained from aortas of diabetic mice exhibit increased levels of inflammatory genes such as *IL‐6*, *MCP‐1*, *MCSF* as well as decreased H3K9me3 levels .^[^ ^67^ ^]^ This data underlines the VSMC inflammatory phenotype transition when subjected to mechanical stimuli.

Lastly, we used CUT&Tag analysis to pinpoint the Piezo1‐driven gene regulation changes.

The application of Yoda1 consistently shown direct regulation of lipid and energy metabolism pathways. Further, the revealed active genes *(klf2, sele, fos*) corroborate Piezo1‐originated atherosclerotic changes.

We find co‐localization of cPLA2 and Piezo1 around the nucleus, as well as lipid droplet formation also in a intimal hyperplasia model. In this model the carotid artery ligation leads to disturbed flow. The disturbed flow causes endothelial dysfunction sustained activation of lipid uptake, synthesis and accumulation.^[^ ^68^, ^69^, ^70^ ^]^ On one hand, this model validates the capability of hsSRS to detect lipid droplet formation in an in vivo model. On the other hand, the change in cPLA2 localization links to our previous data, suggesting an increasing sensitization of VSMC for pressure sensing and/or endothelial‐VSMC signaling, when situated in a compliant extracellular matrix, such as in the hyperplastic intima layer.^[^ ^15^ ^]^ Nevertheless, one limitation of the model is that blood pressure is unaffected by the procedure and does not directly link to hypertensive pressure sensing.^[^ ^71^ ^]^ Moreover, rodents including mice and rats transport only very little cholesterol in LDL, but rather in high density lipoprotein (HDL) like particles.^[^ ^72^ ^]^ Therefore, unless genetically manipulated they are largely resistant to atherosclerosis. While this has no implication on the read out of the in vitro set up, future studies will need to examine the mechano‐lipid metabolic axis in more suitable in vivo models that will also need to consider the cholesterol metabolism.

While key experiments were performed in primary cells, we opted for the A7r5 cell line for the bulk of experiments. Our previous work^[^ ^15^ ^]^ suggested a comparable mechano‐response, while primary human cells showed an early onset of senescence and insensitivity for mechano‐stimulation already after a few passages, thus limiting the number and scale of experiments.

Moreover, the mechanical landscape in the arterial wall is complex and VSMCs are experiencing additionally stresses derived from the dilatation of the arteries, as well as from radial flow.^[^ ^73^, ^74^ ^]^ Our previous work indicated that pressure on its own had a strong effect on the phenotype of the cells, adding to a range of studies demonstrating cellular pressure sensing.^[^ ^15^, ^75^ ^]^ Therefore, our aim here was to study the pressure sensing in further detail. However, mechanosensing of multiple signals (pressure, stretch, stiffness, flow) is likely intertwined and will include additional receptors, transmitter and effector molecules. More advanced in vitro models will be needed in the future to investigate and disentangle the combined effects of the different arterial stresses on the VSMC lipid metabolism and phenotype.

To conclude, this study demonstrates a direct link between the mechanical stimulation and chromatin modification at the histone level, adding to the growing number of studies demonstrating mechano‐epigenetic changes in various diseases.^[^ ^74^ ^]^ This finding is particularly seen in the VSMC – foam cell phenotype transition dependent solely on mechanoregulation. Finally, we propose the Piezo1 channel as a potential therapeutic target in atherosclerosis to counteract the overstimulated lipid metabolism in VSMCs.

## 4 Experimental Section

### Cell Culture

A7r5 rat vascular smooth muscle cell line was obtained from ATCC and cultured in Dulbecco's modified Eagle's medium (DMEM) with 10% fetal bovine serum (FBS), 1% GlutaMAX, and 1% penicillin/streptomycin.

Human VSMCs were obtained by explant culture of thoracic aortic tissue obtained from a healthy female donor aged 35 years (04.35F 11A) as previously described.^[^ ^76^ ^]^ Briefly, 2 mm pieces of tissue were cut from the deep medial layer and placed in a six well plate with minimal media.  A coverslip was placed over the tissue and when explanted cell growth had reached confluence cells were trypsinised for passaging.  Rat VSMCs were obtained using enzyme dispersal of thoracic and abdominal aortas of 12 week old male Wistar rats as previously described.  The tunica adventitia and endothelium were removed by collagenase treatment and the resulting tunica media was dispersed enzymatically as described previously.^[^ ^76^, ^77^ ^]^  Dispersed cells were plated at a density of 8×10^4^ cells cm^−2^. At first trypsinisation both human and rat VSMCs were plated onto coverslips and immunofluorescence was used to establish cell population purity.  All cells were positive for SM‐actin and calponin with no evidence of endothelial cell contamination. Primary cells were used at passages 3–5 cultured in DMEM containing 20% FBS.

### Tissue Sections

8‐week old male mice were subjected to a complete left common carotid artery (LCCA) ligation as described previously.^[^ ^15^ ^]^ 28 days after the procedure, the arteries were collected, 2 mm of proximal as well as distal parts were removed and 4–5 mm LCCA was embedded for obtaining 10 µm thick cryosections on the cryostat microtome (Leica Biosystems).

### Antibodies, Plasmids, and Reagents

Primary antibodies included Piezo1 (Novus Biologicals, NBP‐78446), H3K9me3 (Cell Signaling, 13969S), cPLA (Santa Cruz Biotechnology, sc‐454), GAPDH (Santa Cruz Biotechnology, sc‐47724 for WB), α‐tubulin (Abcam, ab4074 for WB). Fluorescent and HRP‐linked secondary antibodies were from JacksonImmuno Research (Cy2‐Goat Anti‐Rabbit: 111‐225‐144, Cy5‐Goat Anti‐Mouse: 115‐175‐146, HRP‐Goat Anti‐Rabbit: 111‐035‐003; HRP‐Goat Anti‐Mouse: 115‐035‐003). CellROX Green (C10444) and Fluo‐4 AM (F14201) were from ThermoFisher Scientific. Lipidspot (70 065) was purchased from Biotium. The following reagents were used at specified concentrations: Yoda1 (10 µm, Bio‐Techne, 5586/10), Dooku1 (50 µm, Bio‐Techne, 6568/10). Human Piezo1‐1591‐EGFP was a kind gift from Charles Cox. ITPKA9‐dTomato (Tractin‐Tomato) was a gift from M. Schell.

### Immunostaining

Cells were fixed with 4% paraformaldehyde for 10 minutes, permeabilized with 0.5% Triton X‐100 in phosphate‐buffered saline (PBS) for 5 minutes and blocked with 5% bovine serum albumin (BSA) in PBS for 30 min. Cells were incubated overnight with primary antibodies diluted in 1% BSA. The next day, cells were washed in PBS and incubated for 1 hour with secondary antibodies, again washed with PBS and mounted using Prolong Gold (P36930) from Invitrogen. Cells were imaged using Nikon Ti2 SoRa spinning disc microscope with 40x or 60x objectives, using a 1x or 2.8x SoRa disc.

### Western Blotting

Cells were lysed using RIPA buffer (ThermoFisher Scientific) supplemented with proteinase inhibitors (Merck)and phosphatase inhibitor cocktail (Roche). Protein concentration was determined using bicinchoninic acid assay. Samples were resolved on Mini‐PROTEAN TGX precasted gels and overnight transfer was performed at 4°C. Membranes were blocked with 5% BSA, diluted in Tris‐buffered saline with Tween 20 (TBST), and incubated overnight with primary antibodies. Finally, membranes were washed three times with TBST and incubated with secondary antibody for 1 hr. Clarity Western ECL Substrate kit (BIO‐RAD, 1 705 061) was used to develop membranes on the iBright 1500 (ThermoFisher Scientific).

### Real‐Time PCR

A7R5 cells (3.5×10^5^ cells per well) were seeded in a six‐well plate and exposed to DMSO or YODA1 10um for 1 h or 8 h. Total RNA was extracted using the commercial kit RNeasy mini‐Kit (Qiagen, Hilden, Germany). RNA purity and concentration were determined by spectrophotometry using Nanodrop ND‐1000 (Thermo Fisher Scientific).

Total RNA (300 ng) was reverse transcribed using High Capacity cDNA Reverse Transcription Kit (Applied Biosystems, 4 368 814) in a final volume of 25 µL. 5 µL of the cDNA solution were used for the RT‐PCR experiments to measure the amount of CD68 and ABCA1 transcript. StepOnePlus Real Time PCR System (Applied Biosystems) was used to perform the Real‐Time PCR reactions using PowerUp SYBR Green Master Mix (Applied Biosystems, A25742) according to the manufacturer's protocol in a final volume of 12 µL. Primers were purchased from Integrated DNA Technologies (Coralville, USA) and used at 250 nm concentration. Differences in gene expression levels were determined by the 2‐ΔΔCt formula, using GAPDH (glyceraldehyde‐3‐phosphate dehydrogenase) as a reference gene. The following primers were used: CD68 forward 5′‐CTTGGCTCTCTCATTCCCTTA C‐3′, reverse 5′‐TGTATTCCACTGCCATGTAGTT‐3′; ABCA1 forward 5′‐TTGGATTCGGCTGTGAGTATTT‐3′, reverse 5′‐GGACACTGAGGTGGTAAGATTG‐3′; GAPDH forward 5′‐GCAAGGATACTGAGAGCAAGAG‐3′, reverse 5′‐GGATGGAATTGTGAGGGAGATG‐3′; KLF4 forward 5′‐ GCCAGGAGAGAGTTCAGTATTT‐3′, reverse 5′‐ AGCACCATCGTTTAGGCTATTA‐3′;  LGALS3 forward 5′‐ CAACACGAAGCAGGACAATAAC‐3′, reverse 5′‐ GCAAAGTTTCCCGCTCATAAC‐3′; LDLR forward 5′‐ CTGTGGCAGTAGTGAGTGTATC‐3′, reverse 5′‐ CATAGGCACTCATAGCCAATCT‐3′; CD36 forward 5′‐ GCTAGCTGATTACTTCTGTGTAGT‐3′, reverse 5′‐ TTGCCACTTCCTCTGGGTTTT‐3′; MSR1 forward 5′‐ AGGCTGGGTTCTTCAACTTAC‐3′, reverse 5′‐ CCCTGGAGAGATTCCTGATAGA‐3′;

### PDMS Substrates

Polydimethylsiloxane (PDMS) was prepared as described before.^[^ ^78^, ^79^ ^]^ In short, Sylgard 527 was used to obtain the 1 kPa stiffness, pre‐cured to increase the viscosity and then spin‐coated with a 150i spin processor (SPS) onto coverslips and cured overnight at 60C. Before cell plating, PDMS coverslips were plasma‐treated and coated with fibronectin.

### Electroporation

Neon Transfection System (ThermoFisher Scientific) was used to electroporate the A7r5 cells. 3×10^5^ cells were resuspended in a 10 µL buffer and 2ug human Piezo1‐1591‐EGFP and 1ug ITPKA9‐dTomato were added. Two 20 ms pulses at 1100 V were applied and then plated on fibronectin‐coated coverslips.

### Cell Indicators

A7r5 cells were incubated 30 minutes in 37 °C with the respective indicator. Next, cells were washed with a buffer and left to stabilize. Following the baseline recording, Yoda1, or Yoda1 + Dooku 1 were added as a channel specific stimulator and recorded further for another 3–4 minutes. DMSO was used as control.

### Scratch Assay

Wound scratch assays were performed as previously described.^[^ ^80^ ^]^ An equal number of A7r5 cells was plated on glass coverslips. The next day, a confluent cell monolayer was scratched with a sterile 1000ul tip. The cell debris were washed off and new media supplemented with Yoda1 or DMSO was added. After 24 hours at 37 °C 5% CO_2_ the scratched area was assessed. Phase contrast images were taken using Leica DM IL Biological Inverted Microscope equipped with 10x objective lens. Images were quantified as percentage of wound closure using ImageJ.

### Cell Proliferation Assay

Cell proliferation was assessed using EdU‐Click Assay and used according to the manufacturers protocol (Merck, BCK EDU‐488). Briefly, cells were plated on fibronectin‐coated glass coverslips. Treated accordingly and incubated with 5‐Ethynyl‐deoxyuridine (EdU), fixed, stained with DAPI, and imaged using the SoRA Spinning Disc Confocal Microscope.

### TUNEL Assay

Cell apoptosis was assessed using TUNEL assay (Cell Signaling, #25 879) according to the manufacturers protocol. Following the treatment, cells were fixed, incubated with the TUNEL Equilibration Buffer for 5 min and then moved to TUNEL Reaction Mix for 1h at 37 °C. Next, samples were washed 3 times, stained with DAPI and imaged using a SoRa Spinning Disc Confocal Microscope.

### Pressure Chamber

MechanoCulture TR stimulator was used to apply hydrodynamic pressure to A7r5 cells for 30 minutes and 8 hours as described previously.^[^ ^15^ ^]^ Briefly, the stimulator was modified for low‐pressure stimulation in the range of (human) normal (NBP) and hypertensive blood pressure (HT) and perfusion ports to supply pre‐saturated cell culture medium. Cells were stimulated with a sinusoid profile (stretch: 0.5 s, duration: 1 s, hold: 0 s, recovery: 0.5 s) and alternating between set pressures of 16 kPa (peak load) and 8 kPa (pre‐load) to reach a measured pressure profile of 120/60 mmHg for NBP stimulation and between 26 and 16 kPa for a measured pressure profile of 180/120 mmHg for hypertensive pressure stimulation. Control cells were placed inside the stimulator without applying pressure.

### Nanoindentation

Nanoindentation experiments were performed using an Optics11 Chiaro nanoindenter attached to a Leica DMI‐8 microscope, as described previously.^[^ ^15^ ^]^ Cell measurements were performed above the nucleus with an R = 50 µm, k = 0.5 N/m probe (suitable for a stiffness range between ≈0.5 and 80 kPa). The Hertzian contact model was used to fit the data.^[^ ^81^ ^]^ Briefly, the Hertz model is used to obtain the Young's modulus according to the equation:

(1) $$P=\frac{4}{3}{\;E}_{\mathrm{eff}}\;R^{\frac{1}{2}}h^{\frac{3}{2}}$$

whereby 𝑅 is the radius of the spherical indenter tip, ℎ is the indentation‐depth, 𝑃 is load. 𝐸𝑒𝑓𝑓 is the effective Young's Modulus. The Poisson ratio ν relates the Young's modulus to the effective Young's modulus:

(2) $$E_{\mathrm{eff}}=\frac{E}{\left(1-{\;\nu }^{2}\right)\;}$$

whereby an idealized Poisson ratio 𝜈 = 0.5 (for incompressible materials) is used here.

### ELISA Assay

DMSO and Yoda1‐treated samples were lysed using RIPA buffer. S‐Adenosylmethionine (SAM) concentration detection was performed using ELISA kit (Cell Biolabs, MET‐5152) according to the manufacturers protocol. Absorbance (450 nm) of ELISA plates was read using a SPECTROstar^Nano^ micro plate reader (BMG Labtech).

### Transmission Electron Microscopy

Transmission Electron Microscopy (TEM) was performed at the TEM Queen Mary University London Facility. In short, A7r5 cells were fixed for 2 h in 4% glutaraldehyde solution in 0.1 M pH 7.4 Sørensen phosphate buffer. Next the cells were scraped, resuspended in agarose, and cut in 1mm^2^ cubes when set and stained. Ultrathin sections were cut using Reichert‐Jung Leica Ultramicrotome with a Diatome diamond knife. Sections were imaged using JEOL JEM1400 Transmission Electron Microscope, equipped with TEM Morada camera and iTEM (EMSIS) software.

### Stimulated Raman Spectroscopy Imaging

An integrated laser system (picoEmerald S, Applied Physics & Electronics, Inc.) was used to produce two synchronized laser beams at 80 MHz repetition rate. A fundamental Stokes beam (1031.4 nm, 2 ps pulse width) was intensity modulated by an electro‐optic‐modulator with >90% modulation depth, and a tunable pump beam (700–960 nm, 2 ps pulse width, <1 nm (10 cm^−1^) spectral bandwidth) was produced by a built‐in optical parametric oscillator. The pump and Stokes beams were spatially and temporally overlapped using two dichroic mirrors and a delay stage inside the laser system and coupled into an inverted laser‐scanning microscope (Leica TCS SP8, Leica Microsystems) with optimized near‐IR throughput. SRS images were acquired using 40× objective (HC PL IRAPO 40×, N.A. 1.10 water immersion lens) with a 9.75‐48 µs pixel dwell time over a 512 × 512 or a 1024 × 1024 frame. The Stokes beam was modulated with a 20 MHz EoM. Forward scattered light was collected by a S1 N. A. 1.4 condenser lens (Leica Microsystems). Images were acquired at 12‐bit image depth. The laser powers measured after the objective lens were in the range 10–30 mW for the pump beam only, 10–50 mW for the Stokes beam only and 20–70 mW (pump and Stokes beams). The spatial resolution of the system is ≈450 nm (pump wavelength = 792 nm).

Prior to imaging, the plates were aspirated and washed with PBS (2 × 2 mL), the cells were fixed with paraformaldehyde (4% in PBS, 15 min at rt), and washed with PBS (2 × 2 mL). The coverslips were then affixed to glass microscope slides with a PBS boundary between the glass layers prior to imaging following the method described previously.^[^ ^82^ ^]^ For spectral phasor analysis, a wavelength scan was conducted across the range 2800–3050 cm^−1^ (0.4 nm re‐tune, ≈6 cm^−1^, 40 images) from a minimum of three replicate areas containing typically 15 cells per frame for each condition. The image stack was imported into ImageJ and an average intensity projection was created. The spectral phasor analysis was performed as described previously.^[^ ^21^ ^]^ Segmentation of the phasor plot was performed manually using regions‐of‐interest to create images of discrete cellular locations including lipid droplets and cytoplasm regions based on the segmentation outlined in .^[^ ^21^ ^]^ The corresponding average spectra for each ROI is plotted in Origin2018 and normalized between 0 and 1.

### CUT&Tag

Cleavage under targets and tagmentation (CUT&Tag) assay was performed using the CUT&Tag‐IT Assay Kit (Acive Motif, 53 160) according to the manufacturers instruction. In brief, 500 000 cells per sample were collected and bound to the concanavalin A‐coated magnetic beads, incubated for 3 hours RT with H3K9me3 anti‐rabbit primary antibody (Active Motif) and further with guinea pig anti‐rabbit secondary antibody for 1hour RT. Following three washes with the Dig‐Wash Buffer, cells were incubated for 1 hour with pA‐Tn5 RT. Samples were washed three times with Dig‐3000 buffer and resuspended in tagmentation buffer for 1hour at 37 °C. Next, extracted and purified DNA was amplified using PCR. Unique combinations of i5 indexed primer and i7 indexed primer were used for each sample. The libraries were sequenced at the QMUL Genomic Center. For analysis sequences were trimmed using trimmomatic and aligned using bowtie2. Samtools was used to remove mitochondrial reads, for filtering of poor quality reads (MAPQ < 30) and unmapped reads. Peaks were called using Macs2 (broad peaks). Highly reproducible peaks were identified using IDR. ChIPseeker was used to annotate peaks and Clusterprofiler was used to identify enriched KEGG pathways.

### Causal Regulator Analysis

To identify master regulators that can explain the profile of our previously published quantitative proteomic data set^[^ ^15^ ^]^ we opted for a network‐based Causal Reasoning algorithm^[^ ^20^ ^]^ (CBDD R package^[^ ^83^ ^]^) which utilized the MetaBase network and pathway data (Thomson Reuters). Each master regulator received a p‐value from the causal reasoning algorithm as previously described.^[^ ^20^ ^]^

### Quantification and Statistical Analysis

Datasets were tested for normal distribution using the Shapiro–Wilk test. All box plots are displayed as median (central line), upper and lower quartile (box), ±1.5x inter quartile range (whiskers). All experiments were performed as minimum three independent biological repeats, unless otherwise indicated. For determination of the Irreproducibility Discovery Rate (IDR), a 5% cut‐off was selected for the algorithm (IDR rank 0.05;, i.e., peaks have a 5% chance of being an irreproducible discovery). All statistical tests are indicated in the figure legends. For ANOVA tests, Dunnet correction for multiple comparison was applied when comparing to a control condition (i.e., Figure 3, hsSRS) and Tukey correction was applied when comparing each condition with every other condition. All statistical tests were performed with Graphpad Prism.

### Ethics Statement

Human samples were obtained with written informed consent and approval from the Research Ethics Committee, which conforms to the principles outlined in the Declaration of Helsinki. All animal experiments were conducted according to the Animals (Scientific Procedures) Act of 1986 (UK). All the animal procedures were approved by the Queen Mary University of London ethics review board (PPL number: PB508B78D) and conform to the guidelines from Directive 2010/63/EU of the European Parliament on the protection of animals used for scientific purposes or the NIH guidelines (Guide for the Care and Use of Laboratory Animals).

## Conflict of Interest

The authors declare no conflict of interest.

## Supporting information

Supporting Information

Supplemental Table 1

Supplemental Table 2

Supplemental Table 3

Supplemental Table 4

Supplemental Table 5

Supplemental Table 6

Supplemental Table 7

Supplemental Table 8

Supplemental Table 9

Supplemental Table 10

Supplemental Table 11

Supplemental Table 12

Supplemental Table 13

Supplemental Table 14

## Data Availability

The data that support the findings of this study are available in the supplementary material of this article.
