# Supplementary material for: Hypertensive Pressure Mechanosensing Alone Triggers Lipid Droplet Accumulation and Transdifferentiation of Vascular Smooth Muscle Cells to Foam Cells
Source: Adv Sci (Weinh). 2023 Dec 25;11(9):2308686. doi: 10.1002/advs.202308686 (PMC10916670; doi:10.1002/advs.202308686)
Supplement: Supplementary file 1 — Supporting Information [file ADVS-11-2308686-s008.pdf]

ADVANCED SCIENCE
Open Access

Supporting Information

for *Adv. Sci.*, DOI 10.1002/advs.202308686

Hypertensive Pressure Mechanosensing Alone Triggers Lipid Droplet Accumulation and Transdifferentiation of Vascular Smooth Muscle Cells to Foam Cells

*Pamela Swiatlowska, William Tipping, Emilie Marhuenda, Paolo Severi, Vitalay Fomin, Zhisheng Yang, Qingzhong Xiao, Duncan Graham, Cathy Shanahan and Thomas Iskratsch**

Supplementary Figures:

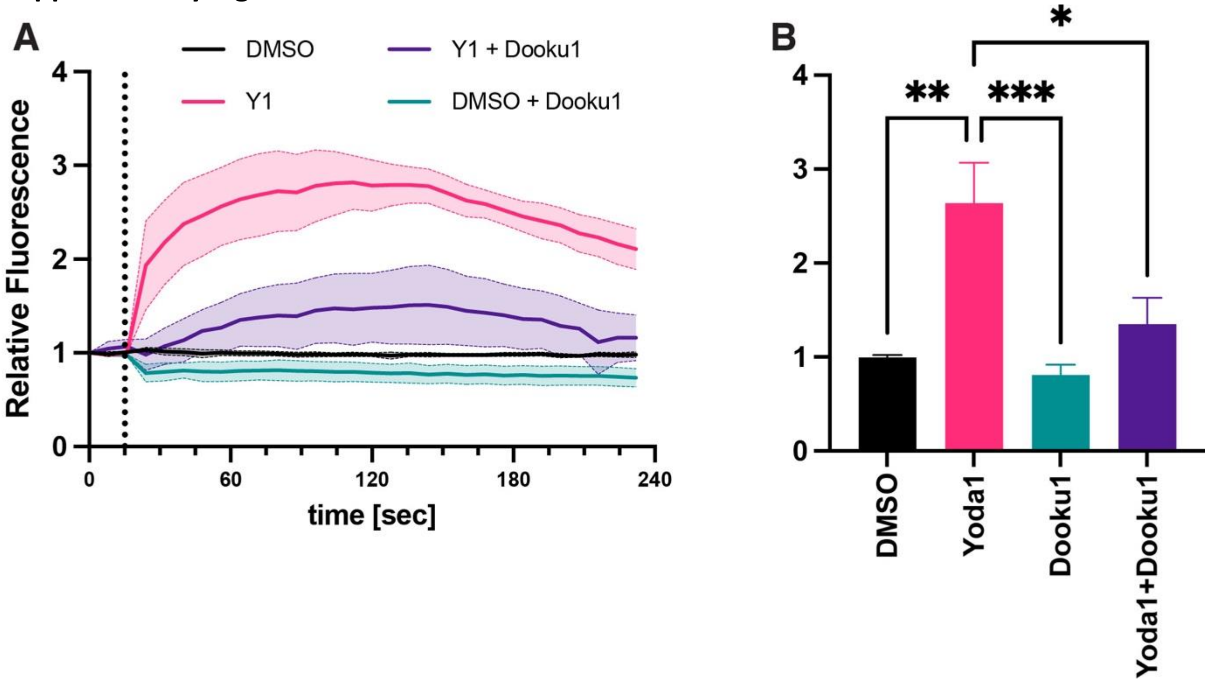

Figure S1. Yoda1 dependent Calcium increase is blocked by Dooku1. A) Yoda1 treatment leads to rapid Ca^{2+} transients in A7r5 that are reduced by simultaneous Dooku1 treatment. Quantified in B. . * p<0.0332, ** p<0.0021, *** p<0.0002; p-values from one-way ANOVA with Tukey correction for multiple comparisons.

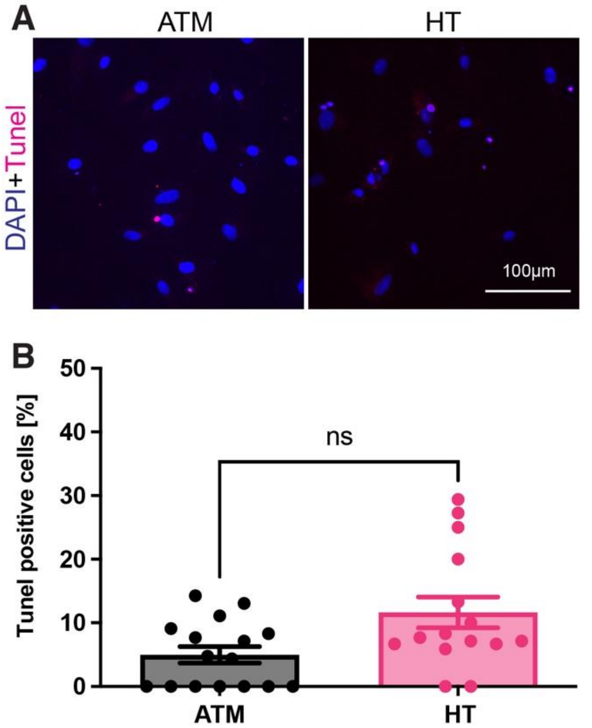

Figure S2. A) Tunel staining indicates no significant changes to apoptosis after hypertensive pressure treatment. Quantified in (B). n=16 and 15 analysed images from three independent repeats. ns: not significant; p-values from Mann-Whitney test.

Hit	Direction	Correct	Incorrect	Unknown	Score	Correctness	Enrichment.Pvalue	Pollard.Pvalue	genename
1	-	42	4	37	38	0.913	2.27E-02	2.55E-09	N-CoR
2	+	41	3	39	38	0.932	1.05E-02	8.09E-10	NF-AT2(NFATC1)
3	+	42	5	36	37	0.894	4.96E-03	1.23E-08	NCOA3 (pCIP/SRC3)
4	-	40	3	40	37	0.930	5.57E-03	1.51E-09	miR-130a-3p
5	-	38	2	43	36	0.950	4.43E-02	7.47E-10	miR-93-5p
6	-	38	2	43	36	0.950	5.32E-03	7.47E-10	miR-429-3p
7	+	41	5	37	36	0.891	7.66E-03	2.2E-08	NCOA1 (SRC1)
8	-	40	5	38	35	0.889	1.93E-02	3.94E-08	miR-24-3p
9	+	38	4	41	34	0.905	1.65E-03	2.83E-08	SENP1
10	+	38	4	41	34	0.905	2.75E-03	2.83E-08	PRMT5
11	-	39	5	39	34	0.886	1.75E-03	7.03E-08	SMRT
12	+	38	4	41	34	0.905	2.09E-02	2.83E-08	NCOA2 (GRIP1/TIF2)
13	+	40	7	36	33	0.851	5.62E-04	5.35E-07	JMJD2B
14	-	39	6	38	33	0.867	5.01E-03	2.71E-07	miR-155-5p
15	-	35	2	46	33	0.946	3.22E-02	5.12E-09	miR-199a-3p

Figure S3. Top 15 outcomes from causal reasoning testing to identify upstream regulators of the of quantitative proteomic data from 1kPa atmospheric pressure vs HT pressure treated A7r5 cells[15]. Yellow shading indicates molecules involved in lipid metabolism and foam cell formation; red shading indicates microRNA hits; grey shading indicates molecules involved in histone modification.

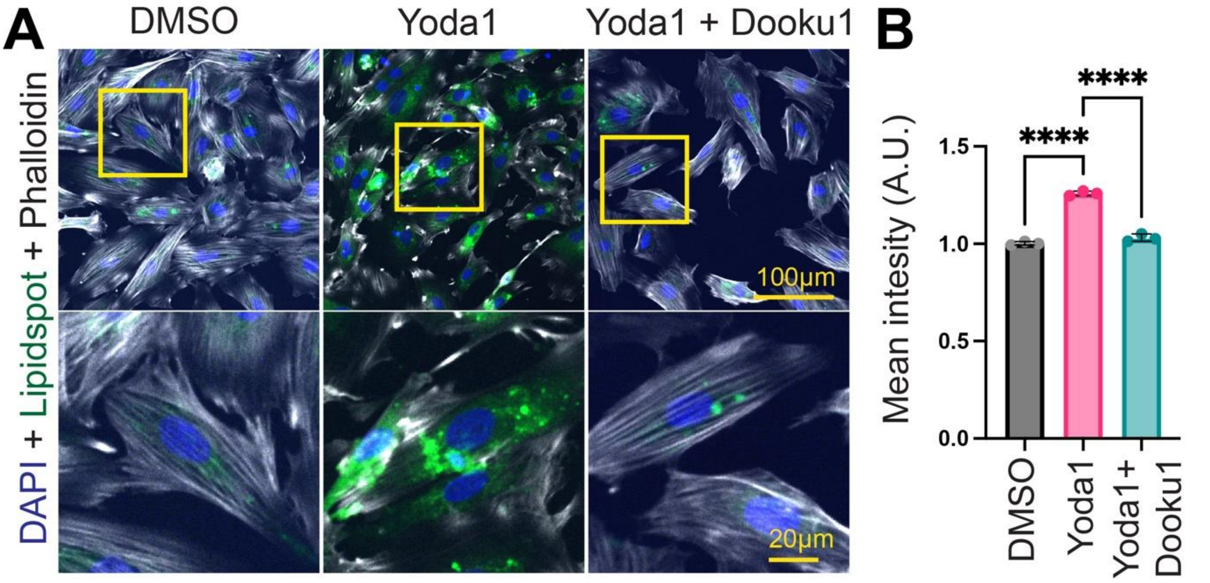

Figure S4. Lipid staining confirms changes in lipid droplets in A7r5 cells. A) A7r5 cells display increased lipid droplet formation after Yoda1 treatment. The lipid droplet formation is reversed when simultaneously treated with Dooku1; B) Quantification from three independent repeats, displayed as mean per repeat, with 250-750 analysed cells per repeat and condition. **** $p<0.0001$; p-values from one-way ANOVA with Tukey correction for multiple comparisons.

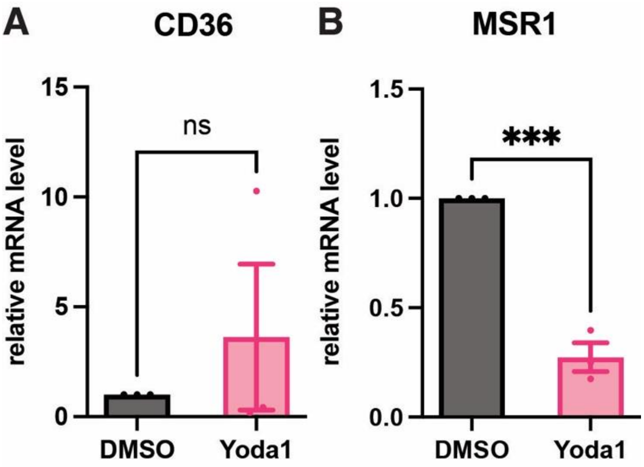

Figure S5. qPCR testing indicate unchanged CD36 and reduced MSR1 levels after 8-hour Yoda1 treatment. p-values from unpaired two-tailed t-tests: ns: not significant, *** p<0.0002.

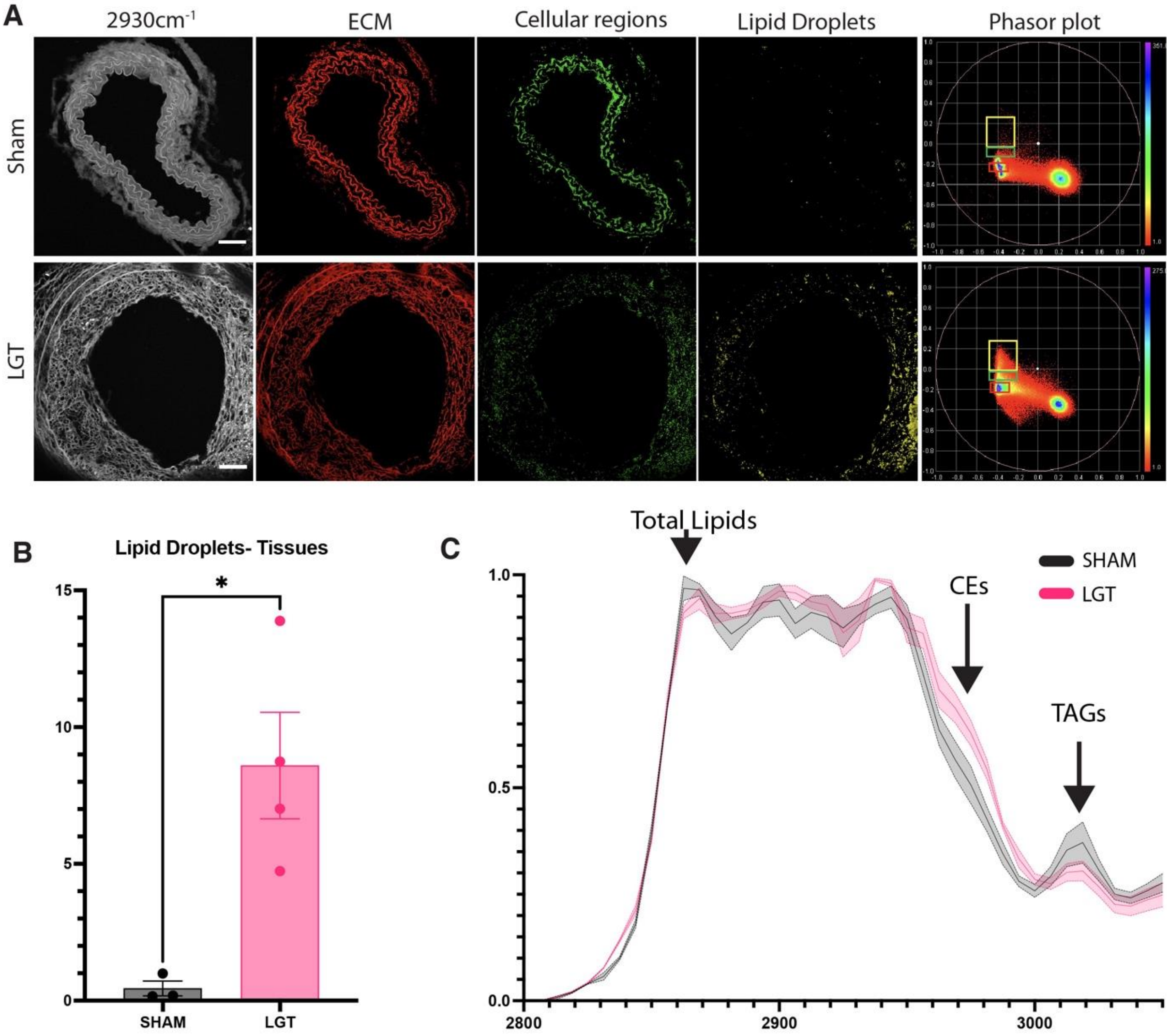

Figure S6. hyperspectral stimulated Raman Scattering (hsSRS) indicates lipid droplet accumulation and lipid metabolic changes during neointima formation. hsSRS was performed on tissue sections after carotid artery ligation (LGT) or in sham operated animals (Sham). A) Example images and segmentation of ECM, cellular regions and lipid droplets from phasor plots (right panels). Lipid droplet formation was increased in LGT arteries (B). C) SRS spectra show increased Cholesterol Esters (CEs) and a reduction in Triacylglycerides (TAGs) in LGT vs Sham control. * p<0.0332; p-values from t-test.

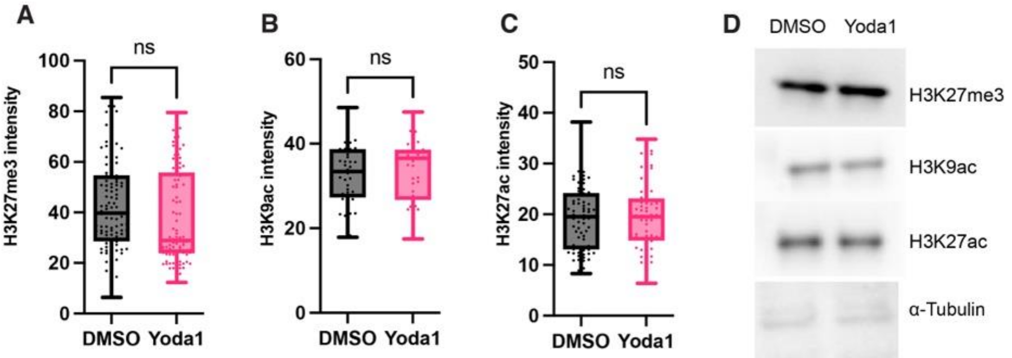

Figure S7. H3K27me3, H3K9ac and H3K27ac histone modifications are unaltered after Yoda1 treatment. A7r5 cells have unaltered H3K27me3 (A), H3K9ac (B) and H3K27ac (C) histone modifications when assessed by immunofluorescence, or western blotting (D). ns: not significant; p-values from unpaired two-tailed t-test.

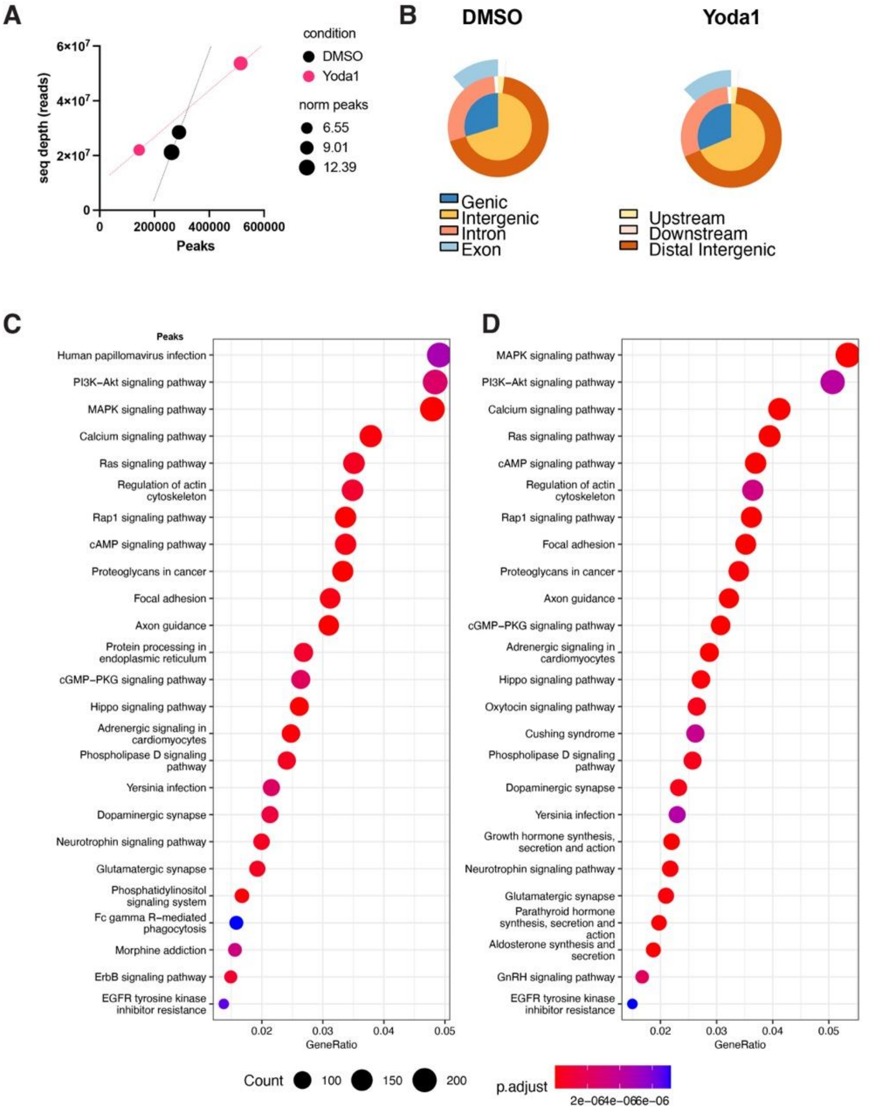

Figure S8. A) Macs2 called CUT&Tag peaks plotted against sequencing depth. Dot size corresponds to the normalised peaks (see also Figure 5). B) Peak analysis indicates comparable positioning of the peaks in relation to genic regions. C) KEGG pathway analysis find overlapping repressed pathways including pathways related to cancer, nervous system, or infections.
